# The Use of Mixed Models for the Analysis of Mediated Data with Time-Dependent Predictors

**DOI:** 10.1155/2011/435078

**Published:** 2011-05-14

**Authors:** Emily A. Blood, Debbie M. Cheng

**Affiliations:** ^1^Children's Hospital Boston and Harvard Medical School, 300 Longwood Avenue, Boston, MA 02115, USA; ^2^Boston University School of Public Health, 801 Massachusetts Avenue 3rd Floor, Boston, MA 02118, USA

## Abstract

Linear mixed models (LMMs) are frequently used to analyze longitudinal data. Although these models can be used to evaluate mediation, they do not directly model causal pathways. Structural equation models (SEMs) are an alternative technique that allows explicit modeling of mediation. The goal of this paper is to evaluate the performance of LMMs relative to SEMs in the analysis of mediated longitudinal data with time-dependent predictors and mediators. We simulated mediated longitudinal data from an SEM and specified delayed effects of the predictor. A variety of model specifications were assessed, and the LMMs and SEMs were evaluated with respect to bias, coverage probability, power, and Type I error. Models evaluated in the simulation were also applied to data from an observational cohort of HIV-infected individuals. We found that when carefully constructed, the LMM adequately models mediated exposure effects that change over time in the presence of mediation, even when the data arise from an SEM.

## 1. Introduction

 In clinical research, both outcomes and predictors are frequently collected repeatedly over time and complex mediated relationships may be present among the variables of interest. For example, in a study of the relationship between alcohol use and HIV disease progression, heavy alcohol consumption may affect antiretroviral therapy (ART) adherence which, in turn, affects CD4 cell count. However, alcohol consumption itself may also directly affect CD4 count. If the goal is to evaluate the total effect of the main independent variable (e.g., alcohol consumption) on the outcome (CD4 count), a single linear mixed effects model (LMM) [1] could be fit to the data. LMMs account for correlation among repeated observations within an individual and are frequently used to analyze longitudinal data. To disentangle the direct versus indirect effects of alcohol use on HIV disease progression, however, a series of LMMs could be fit according to the steps described by Baron and Kenny [2] and demonstrated by Krull and MacKinnon [3] in the mixed model setting. In contrast, if these data were analyzed with a structural equation model (SEM) [4], variables in the causal pathway could be modeled directly by incorporating adherence into the SEM as a mediating variable between heavy alcohol consumption and HIV disease progression. Given the objective is to evaluate the total effect of the main independent variable, it is unclear whether there are benefits to modeling the mediated relationship in terms of bias, coverage, and power for the primary study aim.

Tradeoffs between the use of SEMs and LMMs have been previously evaluated in general settings, and the equivalence of LMMs and SEMs in some settings without mediation has been well documented in the SEM literature [5–12]. The potential advantages of using SEMs over LMMs to analyze longitudinal or hierarchical data include the capacity to explicitly model complex relationships such as mediation [4, 5, 7, 13–16], the flexibility in modeling covariance structures [7, 15], the availability of fit indices [8, 9], and the capability to account for measurement error [5, 9, 10, 15]. One disadvantage is the potential complexity of the SEM model and, therefore, the possibility of model misspecification. In addition, from a practical perspective, the SEM may be less convenient to implement given the need for specialized software. Nonetheless, its flexibility and capacity to directly model variables in the causal pathway make it an appealing modeling technique for mediated longitudinal data.

In the absence of mediation, the type of SEM evaluated in this paper is often referred to as a latent growth curve model [13, 17–20]. Incorporating mediation into a latent growth curve framework has been demonstrated using either a time-invariant mediating factor that influences the latent intercept and slope factors of an outcome trajectory [14] or a time-varying mediator that assumes a parallel growth process in which both the mediator and outcome follow growth trajectories [21, 22]. For both of these approaches, mediation occurs at the random effect level (individual), rather than the observation level and, therefore, cannot vary over time. Modeling mediation that occurs at multiple levels in longitudinal data has been described using separate linear mixed effects models [3, 23, 24]. These multilevel models allow for mediation at the individual as well as observation level, but indirect and total effects are estimated from separate regressions. In the multi-level context, methods for assessing mediation at the observation level have been described with the added complexity that all mediated effects are random [25, 26]. Finally, longitudinal mediation has been described outside of the latent growth curve framework using autoregressive structural equation models [24, 27]. These models assume change over time, where the correlation between observations is not due to underlying random effects (latent intercept and latent slope), but rather results from direct association between an outcome and its value at a previous time point. Autoregressive models are, therefore, not a direct extension of LMMs but represent an alternative approach to model mediated longitudinal data. In this paper, we examine an SEM in which mediation is present at each time point and can, therefore, vary at the observation level. We do not assume that the mediator follows a parallel growth process and assume fixed, not random, effects of the mediator on the outcome. The mediated effects are estimated simultaneously rather than through separate multi-level models.

The performance of LMMs relative to SEMs in a longitudinal data setting with a predictor and mediator both measured only at baseline with longitudinal outcomes has previously been studied [28]. The LMM was accurate and efficient in a variety of settings in estimating the total effect of the main independent variable. The main advantage of the SEM was found to be the ability to simultaneously model the direct and indirect effects of the main independent variable. The objective of this study is to extend this previous work to the setting where the predictor and mediator are both time dependent with fixed effects that change across time.

## 2. Methods

 In the current study, we evaluate the performance of the LMM relative to the SEM in the analysis of mediated longitudinal data with a time-varying predictor and mediator. As an example, we consider a prospective cohort study assessing the effect of heavy alcohol consumption on HIV disease progression [29]. The continuous outcome, CD4 cell count, is denoted by *Y*_*j*_. The main independent variable, heavy alcohol consumption, is a time-varying binary variable denoted by *z*_*j*_; ART adherence, the mediating variable, is a time-varying variable denoted by *M*_*j*_; and baseline age, a continuous covariate, is denoted by *w*. ART adherence is a mediator if the primary independent variable, heavy alcohol use, affects CD4 count indirectly through ART adherence. In addition to indirect effects, heavy alcohol use may also have a direct effect on CD4 cell count that is not mediated by ART adherence or other variables. We focus on a setting where the primary aim is to determine the total effect (direct and indirect effect) of heavy alcohol use on CD4 cell count while appropriately accounting for the mediating effect of ART adherence. We arbitrarily assume there are six time points at which the outcome, predictor, and mediator are measured. Time is represented by *t*_*j*_  (*j* = 1,2,…, 6), and times are assumed equally spaced. In this setting, an LMM could be used to evaluate the total effect of alcohol consumption on CD4 cell count while accounting for correlation due to multiple assessments from the same individual and confounding effects of covariates. Using a LMM would not, however, allow for directly modeling mediation among the variables. SEMs are an alternative approach with the advantage of simultaneous modeling of direct and indirect effects of alcohol consumption on CD4 cell count. The objective of this paper is to evaluate whether the LMM performs adequately relative to the SEM when the goal is to determine the total effect of alcohol consumption, rather than to evaluate whether a variable (e.g., adherence) is a mediator. A series of simulation studies is carried out to evaluate the performance of several LMMs and SEMs under different conditions. We also describe the application of the various models to data from a prospective cohort study evaluating the impact of alcohol use on HIV disease progression.

### 2.1. General SEM Incorporating Mediation

 There are two components to an SEM, the measurement model and the structural model [4]. The measurement model relates unobserved latent variables and covariates to outcomes and exposure indicators. In the measurement model, outcomes are observed variables, while predictors may be observed or latent variables. This model attempts to capture measurement error in observed variables. In the SEM, the repeated observations of CD4 count are the outcomes in the measurement model. The predictors in this model include underlying individual intercept and slope variables as well as time-varying primary independent variable (heavy alcohol use) and the time-varying mediator (ART adherence).

The second component to an SEM, the structural model, models latent variables as a function of observed variables and other latent variables. This model attempts to capture individual variation in the latent variables. In our model, the underlying individual intercept and slope variables are treated as latent variables and modeled as the outcomes of the structural model. In the case of the SEM incorporating time-varying mediators, the repeated mediators (ART adherence), while not latent variables, are also outcomes predicted with some error by the time-varying primary independent variable (alcohol use) so they are incorporated in the structural model.

The general SEM incorporating mediation is described in the following equations. The subject index has been dropped in the equations below for simplicity:

(1) $$Y_{j}=U_{1}+t_{j}U_{2}+\lambda_{j}M_{j}+\kappa_{j}z_{j}+\epsilon_{j},$$

(2) $$U_{1}=\alpha_{1}+\gamma_{2}w+\zeta_{1},$$

(3) $$U_{2}=\alpha_{2}+\zeta_{2}.$$

for *j* = 1 to 6,

(4) $$M_{j}=\alpha_{3}+\gamma_{1j}\;z_{j}+\zeta_{2+j},$$

where var(*ϵ*) = *σ* ^2^ *I* and cov (*ζ*_1_, *ζ*_2_) = Ψ and cov (*ζ*_3_ : *ζ*_8_) = Φ.

The parameters and latent variables in the above equations are interpreted as follows.

- *U*_1_ is the random intercept of the repeated outcomes.
- *U*_2_ is the random slope of the repeated outcomes.
- *λ*_*j*_ represents the effect of the mediator on the outcome at time *j*.
- *γ*_1*j*_ represents the effect of the main independent variables on the mediator at time *j*.
- *κ*_*j*_ represents the direct effect of the main independent variable on the outcome at time *j*.
- The product *λ*_*j*_ × *γ*_1*j*_ represents the indirect effect of the main independent variable on the outcome at time *j*.
- *γ*_2_ represents the constant effect of the continuous covariate on the repeated outcomes.

**Figure 1:**
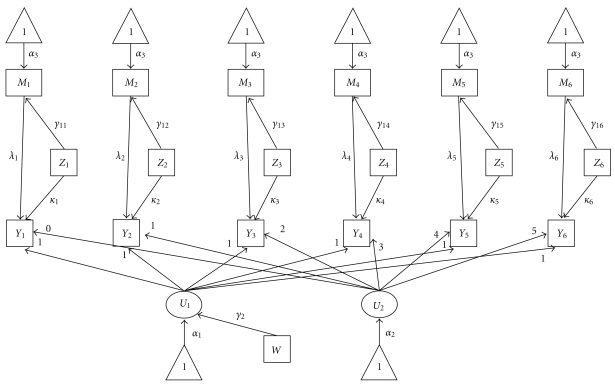
Path diagram of unrestricted structural equation model.

The SEM mediation model is represented in Figure 1. In the following diagram we have used the conventions for SEM path diagrams including rectangles representing observed variables, ovals representing latent variables, triangles representing intercept terms, and arrows representing regression relationships between variables.

### 2.2. SEM Used for Data Generation

 The simulated mediated data for this study are generated from an SEM, because our goal is to evaluate the performance of the LMM in a setting where the SEM is assumed to be optimal. We considered a setting where the effects of alcohol, the main independent variable, changed across time. Specifically, we assumed a constant short-term effect of alcohol on CD4 count for the first three time points and a constant long-term effect of alcohol across the last three time points. We refer to this as a “delayed effect” of the main independent variable. To model this delayed effect, we allowed *κ*_*j*_ in (1) to vary. Specifically, we set the first three *κ*'s to be equal (κ* = *κ*_1_ = *κ*_2_ = *κ*_3_) and the last three *κ*'s to be equal (*κ*′ = *κ*_4_ = *κ*_5_ = *κ*_6_), where *κ*′ > κ*. Short and long-term effects were similarly defined for *λ*_*j*_ and *γ*_1*j*_. Under these assumptions, it can be shown that the predictive formula for a given outcome at time *t*_*j*_, for *j* = 1,2, 3 is

(5) $$\begin{array}{cc} Y_{j} & =\left(\alpha_{1}+\lambda^{*}\alpha_{3}\right)+\gamma_{2}w+\alpha_{2}t_{j}+\left(\lambda^{*}\gamma_{1}^{*}+\kappa^{*}\right)z_{j}\\ & \;+\left(\zeta_{1}+\lambda^{*}\zeta_{2+j}\right)+\zeta_{2}t_{j}+\epsilon_{j}, \end{array}$$

and for *j* = 4,5, 6 is

(6) $$\begin{array}{cc} Y_{j} & =\left(\alpha_{1}+\lambda^{\prime }\alpha_{3}\right)+\gamma_{2}w+\alpha_{2}t_{j}+\left(\lambda^{\prime }\gamma_{1}^{\prime }+\kappa^{\prime }\right)z_{j}\\ & \;+\left(\zeta_{1}+\lambda^{\prime }\zeta_{2+j}\right)+\zeta_{2}t_{j}+\epsilon_{j}. \end{array}$$

The model assumes a linear effect of time on the outcome.

#### 2.2.1. Simulation Procedures

 For the initial set of simulations, we varied the distribution of the total effect of the predictor on the outcome. We evaluated three situations: (i) the total effect was equally distributed between the direct and indirect effect, (ii) the total effect was primarily direct (i.e., the direct effect was larger than the indirect effect through the mediator), and (iii) the total effect was primarily indirect (i.e., the indirect effect through the mediator was larger than the direct effect of the predictor on the outcome).

These simulations considered a setting where the true total effect of the primary independent variable was small (0.05) for the first three time points and small to moderate for the second three time points (0.25), as defined by Cohen [30]. These effect sizes were selected as they are considered feasible and realistic for a wide range of clinical settings. Effect size was defined as the true value of the regression parameter divided by the true standard deviation of the residual error term (*ϵ*_*ij*_). We fixed the true standard deviation of all residual error terms in the simulated data to one, so the effect size is equal to the true value of the regression coefficient. We used a sample size of 350 as this sample size yielded adequate power for the second three time points with the effect size we assumed. 

In addition to the initial set of simulations, we also performed simulations evaluating sample sizes ranging from 100–500 and alternative effect sizes, for example, small negative effect sizes as observed in the example data set described in Section 4, a moderate effect size (0.50) as defined by Cohen [30], and a null effect size to evaluate the Type I error properties of the models.

Model performance with respect to the effect of the primary independent variable on the outcome was evaluated separately for each time-point. 

We generated data using the SEM described above with repeated measures of a continuous outcome, a random intercept and slope and a time-varying main independent predictor and mediating variable. The outcome, main independent variable, and mediator were each assessed at 6 time points. The following steps were taken to generate the mediated longitudinal data.

1. Two multivariate normal random variates were generated, one corresponding to the residual variance of the latent intercept and one to be the residual variance of the latent slope.
2. Six multivariate normal random variates were generated corresponding to the residual variance of the mediator variables.
3. Based on equations (2) and (3), the value of the latent intercept and latent slope were computed.
4. Based on equation (4), the values of the mediator variables were determined.
5. Six independent standard normal random variates were generated corresponding to the residual error of the six *Y*_*j*_'s.
6. Based on equation (1), the value of the *Y*_*j*_'s were generated.
7. Steps (1) through (6) were repeated 1000 times to create 1000 datasets for each simulation.

The models fit to the simulated data were evaluated by assessing: (i) Bias: estimated as the difference between the true parameter value and the mean observed parameter value divided by the true parameter value. (ii) Coverage probability: estimated as the percentage of the 1000 95% confidence intervals that contained the true parameter value. (iii) Power: estimated as the percentage of the 1000 datasets in which a hypothesis test of the parameter of interest was statistically significant and (iv) Type I error: for settings assuming null effects (for both direct and indirect effects), Type I error was estimated as the percentage of the 1000 datasets in which a hypothesis test of the parameter of interest was statistically significant.

### 2.3. SEMs and LMMs Fit to the Simulated Data

 After the simulated data were generated as described above, the data were fit with three SEMs and five LMMs representing a range of possible models that could be fit to mediated longitudinal data.

#### 2.3.1. Constant Effect SEM

 The first SEM we evaluated represents one of the simplest and most common models that can be fit. This model assumes that the direct effect of alcohol on CD4 count is constant (i.e., *κ* = *κ*_1_ = ⋯ = *κ*_6_), the effect of alcohol on ART adherence is constant (i.e., *γ*_11_ = *γ*_12_ = ⋯ = *γ*_16_), and the effect of ART adherence on CD4 count is constant (i.e., *λ*_1_ = *λ*_2_ = ⋯ = *λ*_6_). The total effect of the repeated primary independent variable on the repeated outcome is therefore represented by *κ* + *λγ*_1_. We refer to this model as the constant effect SEM (CESEM).

#### 2.3.2. Delayed Effect SEM

 The second SEM fit to the simulated data is the model that was used to simulate the data and defined in Section 2.2; that is, it assumes an early versus late effect. In this model, a short-term total effect of alcohol on CD4 count (κ* + λ*γ_1_*) is assumed for the first three time points, and a long-term effect of alcohol on CD4 count is assumed for the second three time points (*κ*′ + *λ*′*γ*_1_′).

#### 2.3.3. Unrestricted SEM

 The last SEM evaluated is the unrestricted model defined in (1)–(4) and represented in Figure 1. The unrestricted SEM is a model that could be used to evaluate the nature of a mediated longitudinal relationship between alcohol and HIV disease progression without assuming how the effects may change across time.

#### 2.3.4. Constant Effect Linear Mixed Model

 The first mixed model fit to the simulated data assumes the effect of the repeated primary independent variable to remains constant over time. The formula for this constant effect mixed model is

(7) $$Y_{j}=\beta_{0}+\beta_{1}w+\beta_{2}t_{j}+\beta_{3}z_{j}+b_{1}+b_{2}t_{j}+\epsilon_{j},$$

where var(*ϵ*) = *σ* ^2^ *I* and cov (**b**) = Ψ.

In this model, the interpretation of the parameters is as follows.

- *β*_0_ is the intercept of the repeated outcomes.
- *β*_1_ is the effect of the continuous covariate, *w*, on the repeated outcomes.
- *β*_2_ is the effect of time, *t*_*j*_, on the repeated outcome.
- *β*_3_ is the effect of the repeated primary independent variable, *z*_*j*_, on the repeated outcomes.
- *b*_1_ is the random intercept of the repeated outcomes.
- *b*_2_ is the random slope of the repeated outcomes.

We note that the mediating variable has been excluded from this model, since the goal is to evaluate the total effect of the main independent variable. If a known mediator is included in a model, then the parameter estimate associated with the primary predictor estimates the direct, rather than the total effect, of that predictor on the outcome [28]. Under the constant effect LMM defined in (7), the total effect of alcohol on CD4 count at any time-point is represented by *β*_3_.

#### 2.3.5. Full Delayed Effect Mixed Model

 To capture potential short-term and long-term effects, we allowed the effect of alcohol at the first three time points to differ from that at the last three time points. To accomplish this, an indicator variable representing observations from the last three time points was entered into the model (i.e., indicator variable *I*(*j* > 3) = 1 at time points 4,5 and 6 and *I*(*j* > 3) = 0 otherwise) and the following model was fit:

(8) $$\begin{array}{cc} & Y_{j}=\beta_{0}+\beta_{1}w+\beta_{2}t_{j}+\beta_{3}I\left(j>3\right)\\ & \;+\beta_{4}z_{j}+\beta_{5}I\left(j>3\right)z_{j}+b_{1}+b_{2}t_{j}+\epsilon_{j}. \end{array}$$

Therefore, the regression model for *j* = 1,2, 3 would be

(9) $$Y_{j}=\beta_{0}+\beta_{1}w+\beta_{2}t_{j}+\beta_{4}z_{j}+b_{1}+b_{2}t_{j}+\epsilon_{j},$$

and for *j* = 4,5, 6, it would be

(10) $$Y_{j}=\left(\beta_{0}+\beta_{3}\right)+\beta_{1}w+\beta_{2}t_{j}+\left(\beta_{4}+\beta_{5}\right)z_{j}+b_{1}+b_{2}t_{j}+\epsilon_{j}$$

In this model, the total effect of the repeated primary independent variable is represented by *β*_4_ for the first three time points and *β*_4_ + *β*_5_ for the second three time points. In addition, the intercept of the repeated outcomes is given by *β*_0_ for the first three time-periods and by *β*_0_ + *β*_3_ for the second three time-periods. Thus, this model allows for (a) estimating a potentially delayed effect of alcohol (*z*_*j*_) and (b) accounting for a period effect, by allowing different intercept values for the early and late time periods. The period effect may be induced by the mediator's changing direct effect (in the SEM from which the data are generated, the mediator effect is λ*α_3_ from (5) for the first three time points and *λ*′*α*_3_ from (6) for the last three time points).

#### 2.3.6. Naive Delayed Effect Mixed Model

As described above, the simulated data are generated from an SEM where the effect of the mediator changes over time. In practice, such time dependent effects can be modeled directly as part of the mediation process using SEMs. In contrast, in LMM models, this difference in mean outcome value for early versus late effects can be captured by a time-varying intercept. However, the need for a time-varying intercept term is not immediately clear when fitting a mixed model in this setting, and thus, a model without time-varying intercepts may be more commonly fit. We refer to such a model as the naive delayed effect model

(11) $$\begin{array}{cc} Y_{j} & =\beta_{0}+\beta_{1}w+\beta_{2}t_{j}+\beta_{3}z_{j}\\ & \;+I\left(j>3\right)\beta_{4}z_{j}+b_{1}+b_{2}t_{j}+\epsilon_{j}. \end{array}$$

This model is similar to the full delayed model but assumes the intercept of the repeated outcomes, *β*_0_, is the same for all six time periods. In this naive model, the total effect of alcohol on HIV disease progression is given by *β*_3_ for the first three time points and by *β*_3_ + *β*_4_ for the second three time points.

#### 2.3.7. Time Interaction Linear Mixed Model

 In mixed models, an interaction between time and the main independent variable is commonly included to assess whether the effect of the independent variable changes linearly across time

(12) $$\begin{array}{c} Y_{j}=\beta_{0}+\beta_{1}w+\beta_{2}t_{j}+\beta_{3}z_{j}+\beta_{4}t_{j}z_{j}+b_{1}+b_{2}t_{j}+\epsilon_{j}. \end{array}$$

In this model, the total effect of alcohol (*z*_*j*_) is modeled as a linear function of time, *t*_*j*_, and is represented by *β*_3_ + *β*_4_ *t*_*j*_.

#### 2.3.8. Unrestricted Mixed Model

 The last mixed model we evaluated allowed the effect of alcohol on CD4 count to vary at each time-point, without assuming linearity. The equation for this unrestricted LMM is

(13) $$\begin{array}{cc} Y_{j} & =\beta_{0}+\beta_{w}w+\beta_{t_{j}}I\left(t_{j}\right)+\beta_{z}z_{j}+\beta_{z,t_{j}}I\left(t_{j}\right)\;z_{j}\\ & \;+b_{1}+b_{2}t_{j}+\epsilon_{ij} \end{array}$$

where *I*(*t*_*j*_) is an indicator of time point and is defined as *I*(*t*_*j*_) = 1 if *t*_*j*_ = *j* and *I*(*t*_*j*_) = 0 otherwise. In this model, the effect of *z*_*j*_ is a function of time and is represented by *β*_*z*_ + *β*_*z*,*t*_*j*__ *I*(*t*_*j*_). This is the least restrictive model and is sometimes called a profile analysis [31].

### 2.4. Model Comparisons

To evaluate the performance of the LMM relative to the SEM, we made the following five comparisons.

1. Constant effect SEM (CESEM) versus constant effect mixed model (CEMM).
2. Delayed effect SEM (DESEM) versus full delayed effect mixed model (FDEMM).
3. Unrestricted SEM (USEM) versus unrestricted mixed model (UMM).
4. Delayed effect SEM (DESEM) versus naive delayed effect mixed model (NDEMM).
5. Unrestricted SEM (USEM) versus time interaction mixed model (TIMM).

We simulated data under the SEM defined in Section 2.2. The SEMs were fit as a reference standard to compare with the LMM results, since the objective was to evaluate the performance of the LMM in a setting where the SEM is assumed to be optimal. For comparisons (1), (2), and (3), the main difference between the models is that the SEM explicitly models the mediation, while the LMM does not. All other aspects of the model are the same. Comparison 4 is of interest, because with time-varying mediated data, the naive delayed effect model is commonly fit within the mixed model framework. However, as described earlier, this model does not fully capture the time-varying mediation process, and thus, it is useful to evaluate its performance against the SEM. Comparison (5) is evaluated since a time interaction mixed model is also a common approach in the mixed model framework when a time-varying relationship is suspected. However, it relies on the assumption that the effect of alcohol is a linear function of time. It is, therefore, of interest to compare this model to the unrestricted SEM, which does not assume linearity.

## 3. Results of Simulation Study

### 3.1. Constant Effect SEM versus Constant Effect Mixed Model

 In a setting where the true effect size changed over time, the estimated power to detect the true effect of the primary independent variables on the outcome from a model assuming a constant effect was generally inadequate with a sample size of 350 (≤66% for both the SEM and LMM in all cases) (Table 1). When effects were distributed equally between direct and indirect effects, estimated power was similar for the two models although slightly higher for the SEM (65% versus 62%). The bias estimates for both the CESEM and CEMM were quite large (180% and 171%, resp., for *t*_1_–*t*_3_ and −44% and −45%, resp., for *t*_4_–*t*_6_), overestimating smaller short-term effects and underestimating larger long-term effects as would be expected. The coverage probability was also quite low although for both models, it was higher for the early time points compared to the later three time points. Similar results were observed when effects were primarily direct and also when they were primarily indirect. We note that we deliberately created a small effect at the first three time points to simulate a delayed effect of treatment on outcome and, therefore, did not expect to have adequate power to detect effects at the first three time points with the sample size evaluated. Similar patterns were observed with different sample sizes and effect sizes. Power was markedly lower for sample sizes less than 350 and for the reduced effect sizes.

### 3.2. Delayed Effect SEM versus Full Delayed Effect Mixed Model

The DESEM and FDEMM had similar power to detect long-term total effects independent of whether effects were equally distributed, primarily direct, and primarily indirect (Table 2). With a sample size of 350 and an effect size of 0.25, the estimated power for the last three time points for the DESEM was slightly higher (83%–85%) than for the FDEMM (82%–84%). The bias for both models was low (−0.1%−1.7% and −0.3%−1.4%, resp.) and the coverage probability was high (95% and 94% for the DESEM and FDEMM, resp.). Similar patterns were observed for other sample sizes with the same effect size. For smaller sample sizes (100 and 200), the power dropped to unacceptable levels (32%–63%).

Again, since the magnitude of the effect at the first three time points is small, we did not expect to have adequate power to detect such an effect in either modeling framework with a sample size of 350. In both models, the power to detect the total effect for the first three time periods was substantially lower than that for the last three (10%–13% in the first three time points versus 82%–85% for the second three time points for both models), where the effects were of a larger magnitude. With a sample size of 400, the power remained low to detect a small effect (−0.11, the observed effect size from the real data example standardized by the residual standard deviation) for both models (30% for DESEM and FDEMM). At all sample and effect sizes, results did not differ substantially between modeling frameworks.

### 3.3. Unrestricted SEM versus Unrestricted Mixed Model

 With a sample size of 350, the performance of the USEM and UMM were very similar, regardless of whether effects were equally distributed, primarily direct, or primarily indirect (Table 3). As seen in previous models, the power to detect the effect at the first three time points was low (6%–9%) for both models. For the last three time points, the power to detect the effects was also low for both models (36%–58% for the USEM and 36%–55% for the UMM). The bias, however, was also quite low for both models (−0.08% to 2.9% for the USEM and −2.6% to 2.6% for the UMM). The coverage probability for both models was good (93%–96% for the USEM and UMM) across the different effect distributions. In these models, no specific relationship with time is assumed in the LMM or the SEM, so both models freely estimate the effect of the time-varying main independent variable on time. The cost of this, however, is that several more parameters must be estimated, and therefore, the power to detect effects is reduced. Similar patterns were observed for other sample sizes and effect sizes. 

### 3.4. Delayed Effect SEM versus Naive Delayed Effect Mixed Model

 With the sample size of 350, when the distribution of the effect was equally distributed, the power to detect the total effect for last three time points was very good for the DESEM (83%), the bias was low (−0.1%), and the coverage probability was high (95%) (Table 2). In contrast, for the NDEMM, there was substantial bias (109%) in estimating the total effect for the last three time points. This naive model clearly does not correctly estimate the effect of the primary independent variable on the outcome. Similar trends were observed in the comparison of the two models regardless of how the total effect was distributed, the sample size, or the effect size.

### 3.5. Unrestricted SEM versus Time-Interaction Mixed Model

 Regardless of the distribution of effects, sample size, or effect size, the TIMM and USEM had low power to detect the effect of the repeated primary independent variable on the repeated outcome for the first three time points (Table 3), as expected. With a sample size of 350, power ranged 7%–9% for the USEM and 5%–56% for the TIMM. For the last three time points at this sample size, the USEM had lower bias but also lower power compared to the TIMM. For the TIMM, incorrectly forcing a linear trend resulted in a larger degree of bias. While the TIMM had a large degree of bias at all time points, it has higher power than the USEM at most time points. This increased power relative to the USEM is likely due, at least in part, to fewer parameters being estimated. The higher power and bias of the TIMM relative to the USEM was also observed for other sample sizes and effect sizes (Table 3).

### 3.6. Type I Error Rates

Table 4 shows the estimated Type I error rates for a range of sample sizes. The nominal Type I error rate was 0.05. The Type I error was remarkably similar between analogous SEM and LMM models. Across all models, the observed Type I error rates ranged from 0.030 (CESEM, sample size of 350) to 0.072 (UMM, sample size of 100).

## 4. Real-Data Example: Alcohol and HIV Disease Progression

To demonstrate the application of the various LMMs and SEMs evaluated in the simulation study, we analyzed data from a prospective cohort study evaluating the effect of alcohol use on HIV disease progression. Samet et al. have previously reported the analyses from this longitudinal cohort study [29]. The original analyses combined data from two cohorts (the HIV-ALC and HIV-LIVE cohorts). To illustrate the models evaluated in this paper, we have used data from the HIV-LIVE study and fit the various LMMs and SEMs of interest. For clarity of presentation, we limited the analyses to observations where subjects reported any ART use during followup (*n* = 319) and included only the following key variables: heavy alcohol consumption (yes versus no), the main independent variable, ART adherence (percentage of pills taken in the last three days), the mediator; age, a potential confounder, and CD4 cell count, the primary outcome. Each variable was assessed every six months for up to four years.

The total effect of alcohol consumption on CD4 count was not statistically significant in any of the SEMs or LMMs fit to the data. Estimated total effects are detailed in Table 5. Both constant effect models showed a small negative effect (−3.7 in the CESEM and −3.0 in the CEMM). The delayed effect SEM and LMM showed similar negative effects in the last four time points although the magnitude of effect in the DESEM was slightly larger (−10.3) than that for the DEMM (−4.1). The magnitude of effect at the first three time points was quite small in both delayed effect models but differed in sign in the DESEM (0.41) and DEMM (−2.3) although neither value was significantly different from zero. The unrestricted models generally showed similar results with effects ranging from −41.8 to 5.7 in the USEM and ranging from −15.8 to 9.5 in the UMM. The direction of the estimated alcohol effects were consistent between models with the exception of the third time-point which had a small estimated negative effect in the USEM (−1.4) and a small estimated positive effect in the UMM (6.9); however, neither effect was statistically significant. The magnitude of the effects were similar between the TIMM and USEM. Since a linear effect of time is assumed in the TIMM, however, all effects after time-point 2 are negative, whereas in the USEM, the direction of effects changes between negative and positive. 

## 5. Discussion

 Mixed models are a useful technique to analyze longitudinal data, with time-dependent variables. They can be applied to mediated longitudinal data, and a series of models can be fit to disentangle direct versus indirect effects of an exposure. However, it is unknown whether they perform well relative to SEMs, a method used for mediational analysis. In this paper, we evaluated the performance of the linear mixed model relative to the SEM in the setting of a time-dependent predictor and mediator, where the effects of both change over time.

The main simulation study assumed that the primary independent predictor had a delayed effect on the outcome (i.e., a small effect at the first three time points and a moderate effect at the last three time points). A range of SEMs (constant effect SEM, delayed effect SEM, and unrestricted SEM) and LMMs (constant effect mixed model, naive delayed effect mixed model, full delayed effect mixed model, time-interaction mixed model, and unrestricted mixed model) were fit to the simulated data.

Three comparisons were made between “analogous" models in that the main difference between models was that the SEM explicitly models the mediation, while in the mixed model, the mediator is removed from the model. The analogous models were constant effect SEM versus constant effect mixed model delayed effect SEM versus full delayed effect mixed model, and unrestricted SEM versus unrestricted mixed model. For each of the three comparisons, the SEM and LMM yielded similar results. The power, bias, and coverage probability were all similar when the SEM and LMM were compared. The results from the analysis of data from a prospective cohort study evaluating the impact of alcohol use on HIV disease progression further illustrated the similarity of results from analogous SEMs and LMMs. 

We also considered two comparisons of nonanalogous models. The first comparison was between the delayed effect SEM and the naive delayed effect mixed model. In the SEM framework, mediation can be directly modeled at each time-point, and therefore, the mediated delayed effect of the time-varying predictor is easily incorporated. In the mixed model framework, however, mediation is not directly modeled. Instead, mediators are removed from the model if the goal is to obtain the total effect of the time-varying predictor on the outcome [28]. Therefore, in the mixed model framework, it may not be clear whether a time-varying intercept term is necessary in the model to properly account for the mediated relationship between the predictor and outcome. Our simulations show that the naive delayed mixed model produced extremely biased estimates of both short- and long-term exposure effects, and coverage probabilities were poor. Therefore, although the naive delayed effect mixed model represents a model that may be a natural choice in the mixed model framework, it may not produce valid estimates. To obtain accurate estimates with the mixed model, fitting the full delayed effect model (with time-specific intercept terms) was required. However, as noted earlier, this model may be nonintuitive. This is a distinct disadvantage of the mixed model framework since the model that may be the most natural to fit may result in inaccurate estimates, whereas a natural choice for the SEM is the full delayed effect model, a model which performed relatively well. The second set of nonanalogous models compared the unrestricted SEM and the time-interaction mixed model. These two models reflect a potential difference in the way that time is handled in the two frameworks. In longitudinal data analysis, SEMs incorporate the value of time as a fixed regression coefficient in the measurement model. Treatment of time is usually limited to a linear main effect of time. If some unspecified nonlinear relationship over time between the predictor and outcome is suspected, the most natural way to evaluate this is to leave the relationship between the time-varying predictor and outcome unrestricted and obtain separate estimates at each time-point as is done in the unrestricted SEM. In mixed models, however, interactions between time and other predictors (time invariant or time varying) are frequently incorporated. In our simulation study, the time interaction mixed model had substantially larger bias compared to the unrestricted SEM. Power was generally higher for the mixed model, possibly due in part to the fewer number of parameters being estimated. The difference between the time-interaction mixed model and the unrestricted SEM was also observed in the real-data example. 

In the setting of mediated longitudinal data where exposure effects change over time, the mixed model performed well relative to analogous SEMs. The delayed effect SEM and full delayed effect mixed model had the best performance in terms of bias, coverage probability, and power in modeling the time-specific relationships between variables. It should be noted that in the setting of mediated time-specific effects, the delayed effect SEM, a natural choice for a model within the SEM framework, yielded substantially better results than the naive delayed mixed model, a natural model to choose within the LMM framework. Two other common models that may be fit, the unrestricted SEM and mixed model, both performed well in terms of bias and coverage probability, however, both had lower power due to the relatively large number of parameters being estimated for the given sample size. We note that the results observed in this study may not be generalizable to other settings, for example, scenarios with more complex pathways and relationships between variables could affect the performance of the LMM.

Linear mixed models can perform well relative to SEMs in the analysis of mediated longitudinal data with a time-dependent predictor and mediator. However, care must be taken to identify an appropriate model that adequately accounts for mediator effects, for example, by including time-varying intercepts and excluding variables in the causal pathway. In the specific setting of delayed effect of the time-varying predictor, common models fit within the mixed model framework may not perform adequately in this mediated longitudinal data setting. However, an appropriately specified mixed model can have good performance relative to the SEM in evaluating the overall effects of a time-varying predictor.

## Figures and Tables

**Table 1:** Performance of SEM and linear mixed model assuming total effect of main independent variable is constant when true underlying effects are small for early time points and small to moderate for late time points.

Simulated data scenarios				Constant effect SEM			Constant effect LMM		
Time point	Effect size	Sample size	Effect distribution	Bias (%)	Coverage probability (%)	Power (%)	Bias (%)	Coverage probability (%)	Power (%)
*t*_1_ : *t*_3_	0.05	350	Equal	180	67	65	171	71	62
*t*_4_ : *t*_6_	0.25	350	Equal	−44	56	65	− 45	53	62

*t*_1_ : *t*_3_	0.05	350	Primarily indirect	179	70	60	164	73	55
*t*_4_ : *t*_6_	0.25	350	Primarily indirect	− 44	55	60	− 47	51	55

*t*_1_ : *t*_3_	0.05	350	Primarily direct	173	69	66	169	69	65
*t*_4_ : *t*_6_	0.25	350	Primarily direct	−45	50	66	− 46	49	65

*t*_1_ : *t*_3_	0.05	100	Equal	168	86	24	159	88	22
*t*_4_ : *t*_6_	0.25	100	Equal	−46	82	24	− 48	81	22

*t*_1_ : *t*_3_	0.05	200	Equal	178	80	41	169	81	38
*t*_4_ : *t*_6_	0.25	200	Equal	−44	73	41	− 46	70	38

*t*_1_ : *t*_3_	0.05	400	Equal	175	65	67	165	69	64
*t*_4_ : *t*_6_	0.25	400	Equal	−45	49	67	− 47	46	64

*t*_1_ : *t*_3_	0.05	350	Equal	395	3	97	369	2	94
*t*_4_ : *t*_6_	0.5	350	Equal	−50	16	97	−53	21	94

*t*_1_ : *t*_3_	0.05	400	Equal	−148	73	8	− 137	76	6
*t*_4_ : *t*_6_	− 0.11	400	Equal	−78	63	8	− 83	60	6

Based on 1000 simulated datasets.

**Table 2:** Performance of SEM and linear mixed model assuming delayed effects of main independent variable when true underlying effects are small for early time points and small to moderate for late time points.

Simulated data scenarios			Delayed effect SEM			Naive delayed effect LMM			Full delayed effect LMM		
Time point	Effect size	Sample size	Bias (%)	Coverage probability (%)	Power (%)	Bias (%)	Coverage probability (%)	Power (%)	Bias (%)	Coverage probability (%)	Power (%)
*t*_1_ : *t*_3_	0.05	350	10	95	13	390	23	53	9.6	95	13
*t*_4_ : *t*_6_	0.25	350	−0.1	95	83	109	9	100	−0.3	94	82

*t*_1_ : *t*_3_	0.05*	350	−4.2	95	11	522	6	82	−5.0	95	10
*t*_4_ : *t*_6_	0.25*	350	1.7	95	83	144	0.8	100	1.4	94	82

*t*_1_ : *t*_3_	0.05**	350	2.2	94	11	−234	61	17	2.2	94	12
*t*_4_ : *t*_6_	0.25**	350	−0.7	95	85	633	48	100	−0.7	96	84

*t*_1_ : *t*_3_	0.05	100	−0.2	94	8	−397	68	20	−0.9	94	7
*t*_4_ : *t*_6_	0.25	100	−2.9	94	34	107	58	92	−2.5	94	32

*t*_1_ : *t*_3_	0.05	200	0.2	95	10	−396	45	34	-0.9	95	9
*t*_4_ : *t*_6_	0.25	200	0.8	95	63	109	28	99	0.7	96	61

*t*_1_ : *t*_3_	0.05	400	0.2	94	14	−400	17	60	−1.4	94	12
*t*_4_ : *t*_6_	0.25	400	8.0	96	88	109	6	100	−0.8	94	86

*t*_1_ : *t*_3_	0.05	350	4.2	96	11	−684	0.2	97	4.1	96	9
*t*_4_ : *t*_6_	0.5	350	0.5	95	100	98	0	100	0.09	95	100

*t*_1_ : *t*_3_	0.05	400	0.4	95	11	−148	80	6	0.6	95	10
*t*_4_ : *t*_6_	−0.11	400	1.5	95	30	−90	74	6	2	95	30

Based on 1000 simulated datasets.

Results are from simulated data with total effects equally distributed between direct and indirect effects, except where indicated.

*Total effect is primarily direct.

**Total effect is primarily indirect.

**Table 3:** Performance of unrestricted structural equation model (USEM) and unrestricted and time interaction linear mixed models (UMM and TIMM, resp.) when true underlying effects are small for early time points and small to moderate for late time points.

Simulated data scenarios			USEM			TIMM			UMM		
Time point	Effect size	Sample size	Bias (%)	Coverage probability (%)	Power (%)	Bias (%)	Coverage probability (%)	Power (%)	Bias (%)	Coverage probability (%)	Power (%)
*t*_1_	0.05	350	16	95	8	− 50	93	6	16	96	7
*t*_2_	0.05	350	9.4	94	8	51	94	19	8.6	94	8
*t*_3_	0.05	350	7.4	94	9	153	75	56	4.9	95	8
*t*_4_	0.25	350	−0.6	94	54	−29	81	76	−0.09	94	54
*t*_5_	0.25	350	−0.4	94	48	−8.9	94	73	−0.5	93	48
*t*_6_	0.25	350	−0.08	93	40	11	93	66	−1.0	94	38

*t*_1_	0.05*	350	−4.0	94	8	−71	94	5	−6.3	95	7
*t*_2_	0.05*	350	−9.6	96	7	38	94	15	−9.8	95	6
*t*_3_	0.05*	350	−1.4	95	8	146	78	49	1.3	95	7
*t*_4_	0.25*	350	2.9	95	55	−29	81	70	2.6	94	54
*t*_5_	0.25*	350	2.2	95	49	−7.4	94	71	1.6	94	48
*t*_6_	0.25*	350	0.4	95	36	14	94	66	−0.5	95	36

*t*_1_	0.05**	350	10	95	7	−60	94	6	8.7	95	7
*t*_2_	0.05**	350	−14	93	7	44	95	18	−14	93	7
*t*_3_	0.05**	350	11	94	8	148	75	56	12	94	9
*t*_4_	0.25**	350	−0.4	95	58	−29	81	79	−0.3	95	55
*t*_5_	0.25**	350	0.0	96	50	−8.6	95	77	0.2	96	48
*t*_6_	0.25**	350	−0.2	96	37	12	95	70	−2.6	96	36

*t*_1_	0.05	100	−25	94	7	−26	94	6	−69	93	6
*t*_2_	0.05	100	8.8	94	6	7.2	93	6	36	94	9
*t*_3_	0.05	100	10	96	5	14	95	5	141	90	20
*t*_4_	0.25	100	−2.0	94	20	−2.2	93	20	−31	90	30
*t*_5_	0.25	100	−3.8	94	17	−3.2	94	17	−9.8	95	29
*t*_6_	0.25	100	−2.5	94	16	−2.2	94	16	11	95	26

*t*_1_	0.05	200	8.2	95	7	−60	95	5	7.7	95	7
*t*_2_	0.05	200	1.2	95	7	45	95	13	0.4	95	6
*t*_3_	0.05	200	−7.8	95	7	150	83	34	−9.2	95	6
*t*_4_	0.25	200	4.0	96	38	−29	97	52	4.1	95	36
*t*_5_	0.25	200	−3.0	95	28	−8.1	95	50	−2.6	95	28
*t*_6_	0.25	200	−0.7	94	24	13	95	45	−1.3	94	25

*t*_1_	0.05	400	41	95	8	−65	94	5	2.7	95	7
*t*_2_	0.05	400	−3.2	94	7	41	94	19	−4.7	97	6
*t*_3_	0.05	400	−0.2	95	9	146	74	58	−2.3	96	7
*t*_4_	0.25	400	−1.3	95	60	−30	76	80	−1.7	95	58
*t*_5_	0.25	400	1.2	95	54	−8.6	95	79	1.0	94	53
*t*_6_	0.25	400	−0.5	96	43	12	95	73	−1.9	96	40

*t*_1_	0.05	400	−6	95	7	52	94	14	−7	95	7
*t*_2_	0.05	400	4	97	6	−33	95	8	3	97	7
*t*_3_	0.05	400	2	95	8	−119	82	5	5	95	7
*t*_4_	−0.11	400	0.4	95	17	−52	83	14	0.9	95	17
*t*_5_	−0.11	400	2.5	95	17	−13	95	23	4	95	17
*t*_6_	−0.11	400	1	95	13	26	94	26	2	95	13

Based on 1000 simulated datasets.

Results are from simulated data with total effects equally distributed between direct and indirect effects, except where indicated.

*Total effect is primarily direct.

**Total effect is primarily indirect.

**Table 4:** Type I error rates for mediated structural equation models and linear mixed models at various sample sizes.

Simulated data		Unrestricted		Delayed effect		Constant effect		Time interaction
Time point	Sample size	SEM	LMM	SEM	LMM	SEM	LMM	LMM
*t*_1_	100	0.050	0.050	0.053	0.053	0.05	0.053	0.053
*t*_2_	100	0.064	0.072	0.053	0.053	0.05	0.053	0.065
*t*_3_	100	0.038	0.039	0.053	0.053	0.05	0.053	0.053
*t*_4_	100	0.047	0.051	0.045	0.043	0.05	0.053	0.045
*t*_5_	100	0.048	0.051	0.045	0.043	0.05	0.053	0.041
*t*_6_	100	0.050	0.053	0.045	0.043	0.05	0.053	0.038

*t*_1_	350	0.053	0.053	0.048	0.046	0.030	0.031	0.054
*t*_2_	350	0.044	0.044	0.048	0.046	0.030	0.031	0.047
*t*_3_	350	0.051	0.051	0.048	0.046	0.030	0.031	0.032
*t*_4_	350	0.049	0.049	0.046	0.045	0.030	0.031	0.038
*t*_5_	350	0.038	0.038	0.046	0.045	0.030	0.031	0.046
*t*_6_	350	0.052	0.052	0.046	0.045	0.030	0.031	0.049

*t*_1_	500	0.058	0.064	0.047	0.045	0.054	0.053	0.052
*t*_2_	500	0.048	0.048	0.047	0.045	0.054	0.053	0.049
*t*_3_	500	0.049	0.046	0.047	0.045	0.054	0.053	0.054
*t*_4_	500	0.049	0.050	0.048	0.048	0.054	0.053	0.047
*t*_5_	500	0.051	0.048	0.048	0.048	0.054	0.053	0.048
*t*_6_	500	0.046	0.047	0.048	0.048	0.054	0.053	0.044

Based on 1000 simulated datasets.

**Table 5:** The total effect of heavy alcohol consumption on CD4 cell count from a prospective cohort study of HIV-infected subjects on antiretroviral therapy (*n* = 319) [29]. Longitudinal regression analyses were performed using linear mixed models and structural equation models, and adjusted mean differences (SE) are reported.

SEM				LMM			
Time point	Constant effect	Delayed effect	Unrestricted	Constant effect	Delayed effect	Time interaction	Unrestricted
*t*_1_	−3.7 (9.6)	0.41 (11.6)	−4.8 (19.6)	−3.0 (11.3)	−2.3 (13.8)	0.44 (22.0)	−7.7 (23.9)
*t*_2_	−3.7 (9.6)	0.41 (11.6)	0.13 (20.1)	−3.0 (11.3)	−2.3 (13.8)	−0.41 (18.1)	6.2 (24.1)
*t*_3_	−3.7 (9.6)	0.41 (11.6)	−1.4 (20.4)	−3.0 (11.3)	−2.3 (13.8)	−1.3 (14.7)	6.9 (23.7)
*t*_4_	−3.7 (9.6)	0.41 (11.6)	5.7 (18.9)	−3.0 (11.3)	−2.3 (13.8)	−2.1 (12.3)	0.68 (22.3)
*t*_5_	−3.7 (9.6)	−10.3 (14.0)	−3.4 (20.2)	−3.0 (11.3)	−4.1 (16.5)	−3.0 (11.3)	−4.6 (24.1)
*t*_6_	−3.7 (9.6)	−10.3 (14.0)	3.8 (22.0)	−3.0 (11.3)	−4.1 (16.5)	−3.8 (12.3)	9.5 (25.1)
*t*_7_	−3.7 (9.6)	−10.3 (14.0)	−13.1 (25.9)	−3.0 (11.3)	−4.1 (16.5)	−4.7 (14.8)	−13.6 (30.3)
*t*_8_	−3.7 (9.6)	−10.3 (14.0)	−41.8 (31.6)	−3.0 (11.3)	−4.1 (16.5)	−5.5 (18.2)	−15.8 (35.1)
